# The Health of the Navy

**Published:** 1894-02-17

**Authors:** 


### The Hospital

AN INSTITUTIONAL JOURNAL OF

The Medical Sciences and Hospital Administration,

WITH

AN EXTRA NURSING SUPPLEMENT.

VOL. XV.—No. 386.] [FEB. 17, 1894.

### THE HEALTH OF THE NAVY.

SHAKESPEARE knew the value of beef, and therefore of health, to an Englishman. Not once, nor twice, but many times, he puts it on record that if you want an Englishman to work or to fight you must supply him with fortitude for the inner man in the shape of solid, substantial food, and plenty of it. What did Rambures say to Orleans? "The island of England breeds very valiant creatures; their mastiffs are of unmatchable courage." To which the Constable of France replied, "Just, just; and the men do sympathise with the mastiffs in robustious and rough coming on. . . . Give them great meals of beef, and iron, and steel, and they will eat like wolves, and fight like devils." Now beef is a measure of health, and the capacity for eating it an indicator of British fighting power. The Englishman who is not well enough to eat beef is in no condition to defend his native shores.

With these convictions in his mind at the present juncture in the history of our naval defences, the writer felt it quite an opportune circumstance that the Annual Statistical Report on the Health of the Navy should be put into his hand. Here was an opportunity of finding out the actual condition of the inner defences of bodily vigour and courage of the sailors, who man and practically constitute our first line of defence. For what are our ships with their guns and armour-plates and sailing powers if the sailors who man them have timid hearts and no power of mighty labour and stern, unyielding endurance? One of the most practical things which any of us can do at the present moment is to investigate the health of the Navy; and one of the most important services which scientific medicine can render to England and civilisation generally is to set forth and expound in their full significance the facts of the bodily condition, and therefore of the courage and fighting power of the British sailor.

It cannot be said that the statistical report which is published under Government authority is all that it might be. Statistical indeed it is, very statistical; but it is hardly anything else. Every practical man desires to know all about the diseases of the Navy it is true, but he desires also to know all about its health. He wishes to know, not only how many sailors are unfit for work and fighting, and why they are unfit; but how many also are fit, and what is the degree of their fitness. He desires, moreover, to be fully informed of the measures which are adopted to prevent disease on the one hand, and to secure a splendid vigour of health and fighting power on the other. The mere statistics of disease form a sorry part of the full and complete report on the health of the Navy which the patriotic medical man desires every year to see but cannot obtain.

The question will force itself upon the attention of the medical critic whether it can be really interesting even to the medical officers of the Navy themselves merely to report, almost without comment, such facts as that at the home stations during the year under consideration 1,545 cases of sickness occurred under group A; that of these 30 were cases of cow-pox, 23 of chicken-pox, 98 of measles, 107 of epidemic rose rash, 128 of scarlet fever, 1 of typhus, 90 of simple continued fever, 66 of enteric fever, 5 of dysentery, 827 of influenza, 166 of mumps, and 4 of diphtheria. It is, perhaps, hardly just to say that there are no comments at all on these 1,545 cases of disease, but the comments, such as they are, add no illumination to the subject, and might very well have been spared. The chief object of such remarks as have been made by the compilers of the report seems to have been to trace all the diseases, not to the condition of the vessels or of the general surroundings of the sailors, but to sources quite independent of, and in some instances far away from, the Navy itself. Even the enteric fever, of which there were no fewer than 66 cases, is not admitted to have originated *de novo* on board any of the vessels of the home fleet. "The immediate neighbourhood," says the report, "is probably responsible" in the case of the ships stationed at Devonport, "for the majority of them, as sporadic cases are said to have been not uncommon all over the three towns." Similarly 16 cases reported from Portsmouth are said to have been "mainly acquired in the locality," Fleet-Surgeon William D. Wodsworth, making the suggestion that "enteric fever is almost endemic at Portsmouth, cases occurring all the year round, especially in the districts affected by our men, Kingston and Landport; most of our cases appear to have been contracted in the townships, and in two, at least, of them the symptoms were identical with those of the disease prevailing there at the time."

Now we may say at once, and frankly, that we do not believe in this explanation. The very care which is shown to divert attention from any possible insanitary conditions in the floating homes of our gallant sailors, and the elaborate precision which notes the absolute identity of symptoms in 2 of 66 cases with the symptoms observed in the prevailing epidemic on shore is extremely suspicious. Identical symptoms! Were the cases not all cases of enteric fever, both ashore and afloat? and is it so extremely remarkable that two cases of the same fever afloat should resemble the majority of the cases which were observed on shore? Why all the cases must have been more or less similar; and to quote a special authority to show that two cases were identical is very like a piece of solemn trifling if it is not something very much worse.

What a medical man, who recognises the public duties of private individuals in a democratic country, desires to be absolutely assured of is that the sailors who constitute the first line of ourfighting force are healthy and fit for fighting, and that the floating homes in which their lives are spent are such as not to conserve merely, but to improve their health and fighting powers. But a careful study of the report we are now considering is not reassuring on these points. On the one hand, there seems to be a very much more extensive development of bacillary, that is of preventible, diseases on board our warships than there ought to be; and on the other, there appears to be a desire on the part of those whom the public hold to be responsible to account for that development on other grounds than those which are probably the true ones. As a matter of fact, such diseases as typhoid, simple continued fever, scarlet fever, and the like ought to be almost unknown in our large and expensively equipped men-of-war. If a large ocean-going passenger steamer is properly looked upon by the public as a floating sanatorium how much more ought a huge warship on whose construction no cost has been spared, and which carries no human beings at all save those who are necessary to her proper working in peace or war, to be a model and an object lesson in all that is sweet and wholesome, and invigorating to mind and body! The point we desire to insist upon is that our sailors, if not healthy, must be made so. We are mad if we entrust our first line of defence to men of whom no fewer than 934·39 per thousand are stated by the Report to have required medical treatment in 1892. Of a total of 58,330 sailors of all ranks and ages, 54,503 were on the sick list for more or less serious disease in that one year.

Is the Navy really so unhealthy as, according to this Report, it appears to be? We are inclined to think that a fuller and more skilful report might have put a better face on the facts. Nevertheless, there is little doubt that our sailors as a whole are by no means the bright, cheerful, healthy, beef-eating, and war-breathing fire-eaters they ought to be. But if that be so, who is responsible? The medical officers of the Navy ought perhaps to be responsible; but are they? We are inclined to think not! It is, we fear, with the Navy as it is with the Army—the combatant officers assume all authority in every department: the medical officers are merely tolerated by their combatant *confrerès*, and that with the worst possible grace. To initiate reforms in the services which in any way come into collision with the established prejudices of unscientific and uncomprehending combatant officers is to stir up, not so much a nest of hornets as a fleet of torpedoes. This is fatal to health and fighting power. Some of us must charge ourselves with the duty of compelling investigation and ensuring reform. It is absolutely imperative that the country shall be able to rely upon the vigorous health and fighting power of its sailors, as well as upon the efficiency of their ships and arms.

